# Benzylaminoethyureido-Tailed Benzenesulfonamides: Design, Synthesis, Kinetic and X-ray Investigations on Human Carbonic Anhydrases

**DOI:** 10.3390/ijms21072560

**Published:** 2020-04-07

**Authors:** Majid Ali, Murat Bozdag, Umar Farooq, Andrea Angeli, Fabrizio Carta, Paola Berto, Giuseppe Zanotti, Claudiu T. Supuran

**Affiliations:** 1 Dipartimento Neurofarba, Università degli Studi di Firenze, Sezione di Scienze Farmaceutiche, Polo Scientifico, Via Ugo Schiff 6, Sesto Fiorentino, 50019 Florence, Italy; hm.ali6584@gmail.com (M.A.); andrea.angeli@unifi.it (A.A.); fabrizio.carta@unifi.it (F.C.); claudiu.supuran@unifi.it (C.T.S.); 2 Department of Biomedical Sciences, Università di Padova, Via Ugo Bassi 58/B, 35131 Padua, Italy; paola.berto@unipd.it; 3 Department of Chemistry, COMSATS University Islamabad, Abbottabad Campus, KPK 22060, Islamabad 45550, Pakistan; umarf@cuiatd.edu.pk

**Keywords:** carbonic anhydrase: sulfonamide, tail approach, X-ray crystallography

## Abstract

A drug design strategy of carbonic anhydrase inhibitors (CAIs) belonging to sulfonamides incorporating ureidoethylaminobenzyl tails is presented. A variety of substitution patterns on the ring and the tails, located on *para*- or *meta*- positions with respect to the sulfonamide warheads were incorporated in the new compounds. Inhibition of human carbonic anhydrases (hCA) isoforms I, II, IX and XII, involving various pathologies, was assessed with the new compounds. Selective inhibitory profile towards hCA II was observed, the most active compounds being low nM inhibitors (*K*_I_s of 2.8–9.2 nM, respectively). Extensive X-ray crystallographic analysis of several sulfonamides in an adduct with hCA I allowed an in-depth understanding of their binding mode and to lay a detailed structure-activity relationship.

## 1. Introduction

Carbonic anhydrases (CA, EC 4.2.1.1) are a ubiquitous superfamily of metalloenzymes present in all living organisms. So far fifteen carbonic anhydrase isozymes have been characterized in humans and they all mainly differ for their catalytic activities, subcellular localizations and tissue distributions [1,2,3,4]. The abnormal expression of one or multiple CA isoforms in humans usually is associated to various pathologies [4,5,6]. Therefore the use of proper CA modulators (i.e., traditionally CA inhibitors, CAIs) represented the winning strategy for the management of various diseases such as glaucoma, altitude sickness, epilepsy and obesity [4,5,6]. A contribute of this kind is the recently reported sulfonamide CAI, named SLC-0111, which entered Phase II clinical trials in association with Gemcitabine for the treatment of metastatic pancreatic ductal cancer in patients overexpressing the CA IX isoform [7,8,9,10]. New evidences in the field strongly support the involvement of CA isoforms in other pathologies, such as neuropathic pain, arthritis, cerebral ischemia and thus pave the way for the use of CAIs in their management [11,12,13,14].

CAs structural architectures have been intensively studied mainly by the use of X-ray crystallographic experiments [15]. They all report quite similar features such as the conical shaped cleft, which bears the catalytic metal ion at the bottom end [15]. As for the human expressed CAs, which are considered in this manuscript, the catalytically active isoform all share the same enzymatic cluster formed by a Zn (II) ion tetrahedrally coordinated to three His-residues and a hydroxide ion/water molecule [16,17,18,19,20].

All clinically used CAIs, which mainly belong to the sulfonamides or sulfamate classes, do present modest and general undesired side-effects after administration, which usually are ascribed to the non-selective interference with CAs other than the desired one/ones [4]. In this contest the “tail approach” proved to be the most successful strategy within the field of Medicinal Chemistry on targeting specific CA isoforms. Such an approach makes use of the interactions occurring between the chemical moieties inserted in the CAIs tails and the amino acid residues located at the rim of the enzymatic cavity, which have the lowest homology sequences among the CA isoforms of the α-class [17,21,22,23,24,25,26,27]. An important structural consideration is the role of the linker connecting the tail and the warhead sections within CAI molecules in order to make use of the tail strategy. The ureidic moiety was demonstrated to be particularly suitable as it allows the entire molecule to assume the best conformation when allocated within the enzyme cavity and at the same time to contribute to the stabilization of the enzyme–inhibitor complex by means of hydrogen bonds [8,9,10,28,29,30]. Subsequently, various CAI moieties bearing ureido linkers were reported [28,29,30,31,32,33,34]. Noteworthy are the *N*’phenyl-*N*-hydroxyureas, the *N*-aryl-*N*′-ureido-*O*-sulfamates [14,35,36] in which the ureidic tether was merged into the CAI moiety or alternatively molecules having the ureido moiety being part of tail section such as the GABA or the *N*-substituted piperazinyl moiety [31,32,37].

In this study we further extended our research by means of designing and synthesizing sulfonamide based CAIs possessing the benzylaminoethylureido-tails, not reported yet, and we investigated their inhibition profiles in vitro against the physiologically dominant cytosolic hCA I and II and the tumor associated isoforms hCA IX and XII with the aim to set the basis for a preliminary structure-activity relationship (SAR) worthy of future development.

## 2. Results and Discussion

### 2.1. Chemistry

**Scheme 1:**
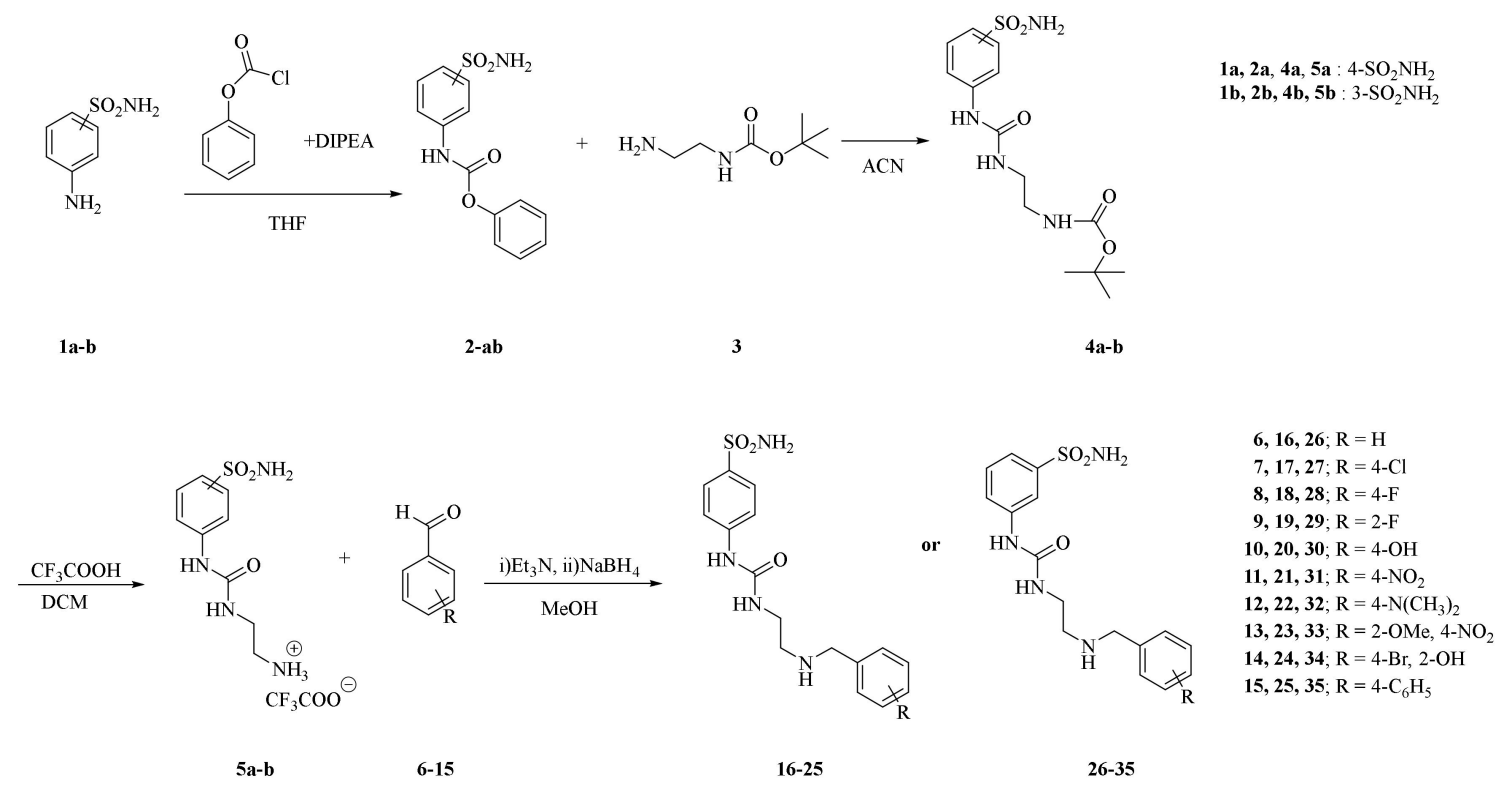
General synthetic scheme for the synthesis of sulfonamides **16-35**.

Our drug design strategy was based on a multistep synthesis, based on the methodology previously reported for obtaining 4-*N*-substituted piperazinyl-ureido benzene sulfonamides [32]. Sulfanilamide (**SA, 1a)** and metanilamide (**MA**, **1b)** were reacted with phenylchloroformate in mild conditions to afford the phenylcarbamate derivatives **2a**–**b** in high yields and purities, in agreement with previous literature reports [32]. **2a**–**b** were coupled with *N*-boc-ethylene diamine **3** to afford Boc-aminoethylureidobenzene sulfonamide derivatives **4a**–**b** in high yields. Then Boc-protecting groups were removed by trifluoroacetic acid to obtain the key intermediates possessing terminal primary amino groups **5a**–**b**. The final step involved reaction of **5a**–**b** with a series of commercially available benzaldehydes **6**–**15** to form the corresponding Schiff bases, thereafter reduced in situ with sodium borohydride to afford the desired products **26**–**35** (Scheme 1).

### 2.2. Carbonic Anhydrase Inhibition

The inhibitory activity of the novel sulfonamides **16-35** on four physiological relevant human (h) CA isoforms; hCA I, II (cytosolic isoforms) and hCA IX and XII (tumor associated membrane bound) reported here was obtained by means of the stopped flow CO_2_ hydrase assay [38]. Inhibition constants of reported compounds and reference drug acetazolamide (**AAZ**) are shown in Table 1. 

The following structure activity relationship (SAR) can be observed from the data reported in Table 1.

- hCA I was inhibited by all these sulfonamides with a variety of potencies. The most potent inhibitor was the 4-F substituted SA derivative **18** with a *K*_I_ of 24.6 nM. Followed by 4-Br, the 2-OH substituted SA derivative **24** showed medium potency with a *K*_I_ of 138.4 nM. The remaining SA derivatives **16**–**18**, **20**–**23** and **25** were weak inhibitors with *K*_I_s spanning between 526.9 and 1506 nM. The MA derivatives of the series **26**–**35** were poor to ineffective inhibitors of the cytosolic widespread hCA I. **26**–**32** and **34** were weak inhibitors with *K*_I_s spanning between 653.8 and 6250 nM. **33** and **35** showed ineffective inhibition of hCA I whereas clinically used drug **AAZ** was medium potency inhibitor with a *K*_I_ of 250 nM (Table 1).
- The series of sulfonamides **16**–**35** showed nanomolar range inhibitory potencies on the physiologically dominant cytosolic isoform hCA II with *K*_I_s spanning between 2.8 and 564.9 nM. The compounds **18**–**19**, **24**–**25**, **27**–**29** and **34** were effective inhibitors with *K*_I_s in the range of 2.8–63 nM. The most effective inhibitor of the series was 4-Br, 2-OH substituted MA derivative **34** with a *K*_I_ of 2.8 nM, also its SA derivative **24** was the third most effective inhibitor reported here with a *K*_I_ of 5.3 nM. 2-F derivative of SA **19** was the second most potent inhibitor with a *K*_I_ of 3.9 nM and its MA derivative **29** inhibited this isoform effectively with a *K*_I_ of 53.8 (8.6-fold less potent). When the fluorine substitution pattern of **19** shifted from the *ortho*- to *para*- position to afford **18** resulted as a slight reduction of the inhibition constant (0.4-fold) to 9.2 nM. MA derivative **28** was a less effective inhibitor compared to its SA counterpart **18** with a *K*_I_ of 53.8 nM (5.8-fold less potent). The last high potency inhibitor of this isoform was 4-Cl substituted MA derivative **27** with a *K*_I_ of 36.9 nM whereas its SA warhead possessing derivative **17** showed medium potency with 3.4-fold decrease compared to **27** with a *K*_I_ of 126.9 nM. SA derivatives **21**–**23**, **25** and MA derivatives **26**, **33** were medium potency inhibitors with *K*_I_s spanning between 63.2–96.1 nM and 76.3–90.2 nM, respectively. The remaining SA derivatives **16**, **20**, and MA derivatives **30**–**32**, **35** were weak to ineffective inhibitors of hCA II with *K*_I_s of 267–350 nM and 166.4–564.9 nM, respectively.
- The tumor associated transmembrane isoform hCA IX was effectively inhibited only by two SA derivatives with fluoro substitutions, **18** in–para and **19** in–ortho positions with *K*_I_s of 30.1 and 20.3 nM, respectively. Their inhibition levels are in comparison with clinically used drug **AAZ** (*K*_I_ 25.8 nM). Compounds **17** and **21**–**24** were medium potency inhibitors with *K*_I_s spanning between 115.6 and 458.3 nM. The remaining ones **16**, **20**, **25**, **27**, **28** and **30**–**34** were low micromolar *K*_I_ values obtained and comprised between 0.97 and 4.21 µM. The remaining of the series **26**, **29** and **35** were not inhibitors of the hCA IX.
- The second extracellular tumor associated isoform hCA XII was inhibited by **16-35** more effectively (except **18** and **19**) compared to the other tumor associated isoform hCA IX investigated here. The 4-bromo-2-hydroxyphenyl substituted derivative **35** was the most potent inhibitor of this isoform with a *K*_I_ of 7.2 nM. **35** showed a comparable *K*_I_ with **AAZ** (*K*_I_ of 5.7 nM). Compounds **16-21**, **23**–**25**, **28**, **31** and **32**–**33** were potent inhibitors with *K*_I_s between 32 and 97.6 nM. Compounds **22**, **27** and **30** showed medium potency inhibition of this isoform with *K*_I_s spanning between 228.1 and 317.1 nM whereas compounds **26**, **29** and **35** showed lower inhibitory effects with inhibition constants between 478.8 and 772.1 nM. The comparison of two subset reported here showed us the derivatives of **SA** were more potent inhibitors versus **MA** derivatives. Whereas 4-nitro substituted **31**, 4-dimethylamino substituted **32** and 4-bromo-2-hydroxy substituted **34** were more potent compare to their **SA** derivatives (1.3, 3.4 and 13.6-folds, respectively). 4-nitro-2-methoxy substituted derivatives **23** and **33** showed very similar inhibition activities on this isoform with *K*_I_s of 53.6 and 53.1 nM, respectively.

Overall, the series showed selective inhibition of the cytosolic isoform hCA II and the membrane bound isoform hCA XII over the hCA I and the tumor associated hCA IX. In particular compounds **18**, **21**, **23**–**25**, **28**, **33** and **34** were the most representative of such a kinetic profile.

### 2.3. Structure of hCA I/Inhibitor Complexes

The crystal structure of six different inhibitors in the complex with hCA I was determined at a resolution ranging from 1.37 to 1.66 Å. Two monomers virtually equivalent (the r.m.s.d. between monomers A and B ranges from 0.197 to 0.25 Å) were present in the asymmetric unit. In all structures, the polypeptide chain was clearly visible from residue 4 to 260 in both monomers. The r.m.s.d. between the monomers A and B of our structure at the highest resolution and that of the apo enzyme in a different crystal form was 0.35 Å and 0.32 Å, respectively, indicating that the binding of the ligand did not induce any significant conformational differences in the protein.

**Figure 1:**
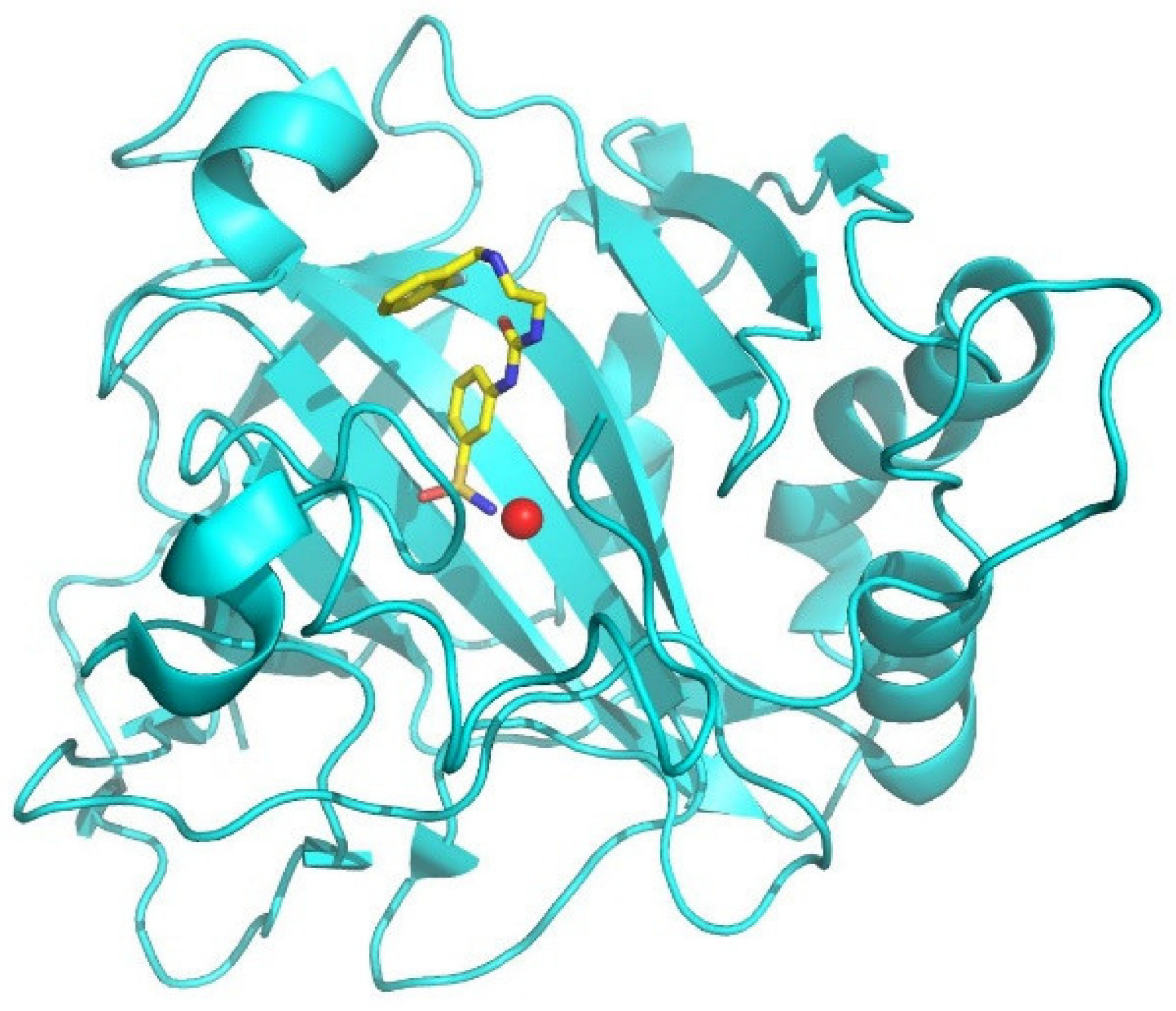
Ribbon drawing of the monomer of hCA I (cyan) present in the asymmetric unit of the P2_1_2_1_2_1_ space group, with compound **29** (PDB code: 6Y00) in the active site. The Zn^2+^ ion is shown as a red sphere.

In all complexes the inhibitor was bound in a similar way, with the nitrogen of the benzenesulfonamide group interacting with the Zn^2+^ ion present in the active site. The coordination of Zn^2+^ ion is tetrahedral, with three corners of the tetrahedron corresponding to three histidines of the enzyme (His 94, 96 and 119), and the fourth corner to the N atom of sulfonamide (Figure 1). In addition, an oxygen atom of sulfonamide forms a H-bond with the main-chain nitrogen of Thr199. In all cases, the benzenesulfonamide group was clearly visible in the electron density map and was oriented in the same way in all six complexes, whilst significant differences were observed in the rest of the inhibitor molecule. In particular, the amino-ethyl-ureido chain was less clearly visible in some of the complexes, indicating a flexible conformation in the crystal.

**Figure 2:**
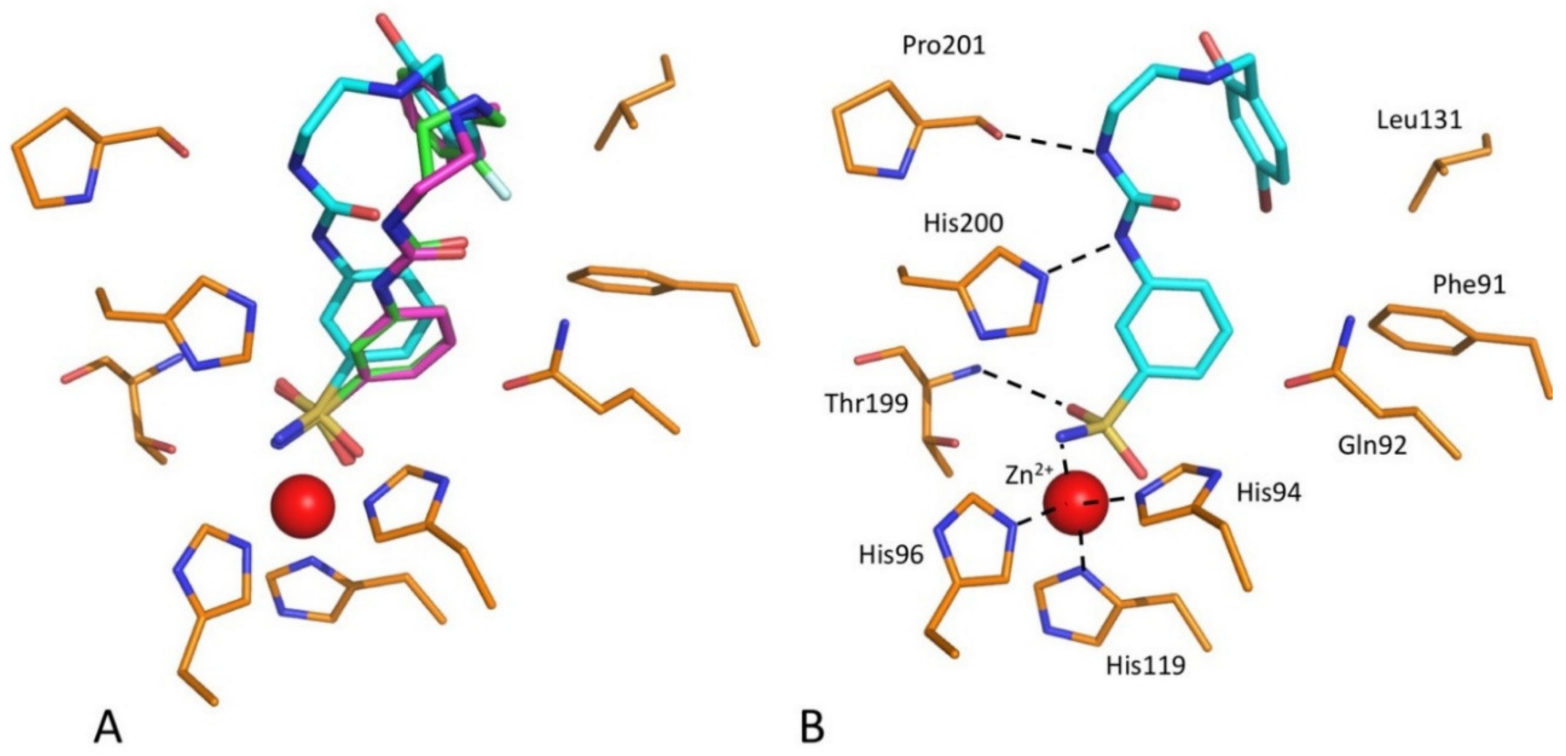
(**A**) Conformations of compounds **26** (magenta, PDB accession code 6XZX), **29** (green, PDB accession code 6XZY) and **34** (cyan, PDB accession code 6Y00) in the active site of monomer B. The conformation and the interactions with the protein are essentially the same for monomer A. Residues surrounding the ligand are shown. (**B**) The active site of compound **34**, with interactions explicitly shown. In both figures the Zn^2+^ ion is depicted as a red sphere.

In the 3-benzene substituted compounds, in the three structures determined that the ligand bound assumed a bent conformation (Figure 2A) and the benzylamino group was accommodated in the hydrophobic portion of hCAI catalytic site. In **34** (Figure 2B) structure, N11 and N14 formed a potential H-bond with Ne2 of His 200 and with carbonyl oxygen of Pro 201. In compound **26**, the carbonyl oxygen formed a H-bond with a side chain nitrogen of Gln 92, whilst in compound **29** the amino-ethyl-ureido chain did not form any specific H-bond interaction with the protein.

**Figure 3:**
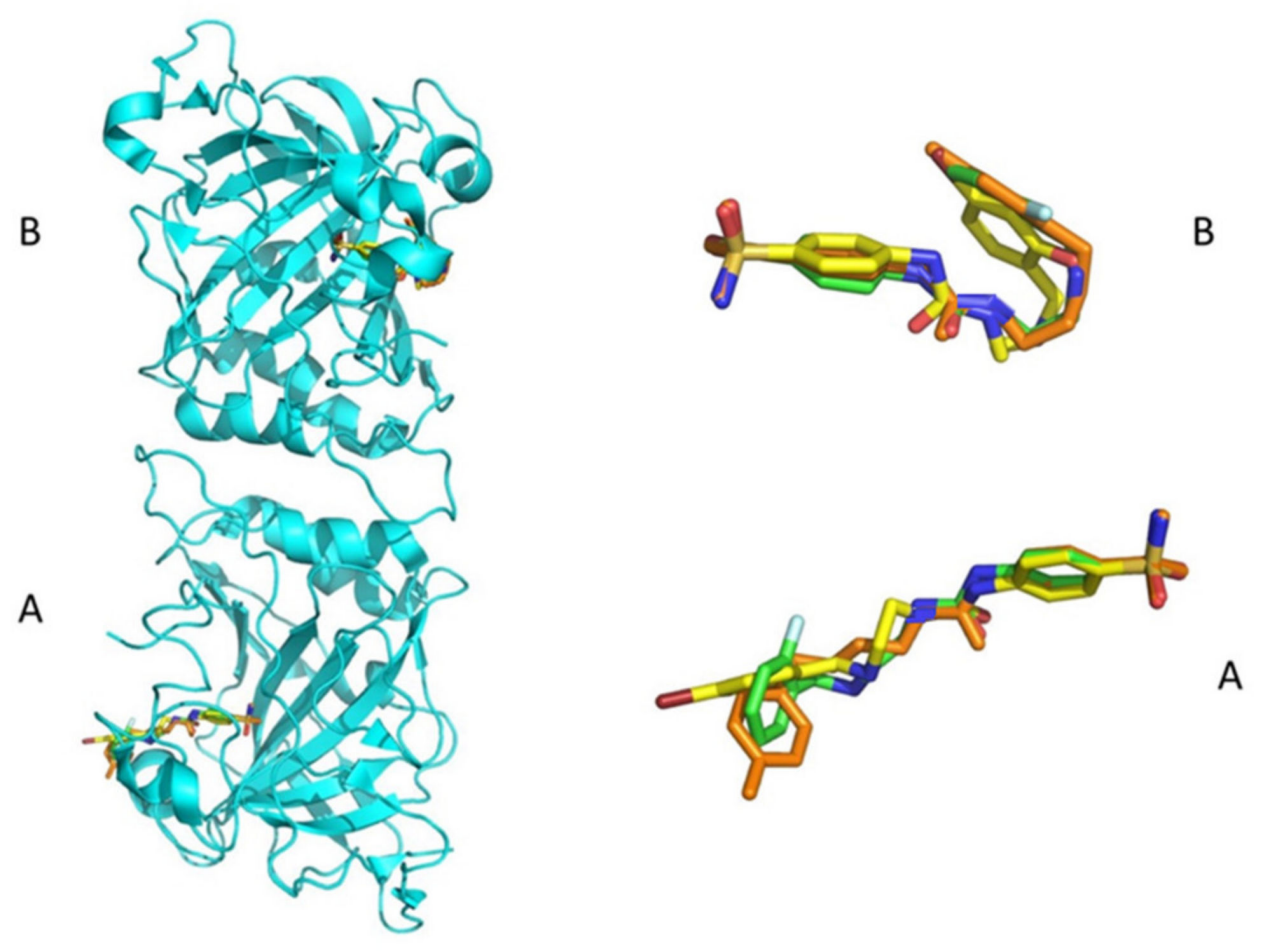
Cartoon view (**left**) of the two monomers of hCA I with compounds **18** (orange, PDB access code 6XZS), **19** (green, PDB access code 6XZE) and **24** (yellow, PDB access code 6XZO) in the active sites of the enzyme. Compounds **18**, **19** and **24** are present in two different conformations in the two active sites of the enzyme (the two sites differ only for the environment in the crystal, but they are conformationally identical, and so they are in solution). In site A the compound is present in an extended conformation, (better illustrated in the right side), whilst in site B the same conformation is not possible owing to the steric hindrance generated by the presence of the same compound in site A, and the compounds folds in a way similar to compounds **26**, **29** and **34** (as in A, the right side of B shows the folded conformations. See text for more details).

In the 4-benzene substituted inhibitors, the geometry of the binding of the benzenesulfonamide group and the interactions with the enzyme were identical to those described above. On the contrary, compound **24** was present in the active site in a nearly extended conformation (Figure 3A). Since the complexes were obtained by soaking the compound inside a solution containing the crystals, in the complex containing compound **24** only one ligand was present in the active site of the two monomers, since, owing to the crystal packing, the compound protruded from the active site in the solvent and partially obstructed the binding of the same compound in the active site of the second monomer. Consequently, the occupancy of each of the two ligands in this case was close to 0.50.

In compound **18** and **19** complexes, the ligand present in molecule B of the crystal assumed a bent conformation, similarly to the 3-substituted ones, whilst they presented a more extended conformation in site A (Figure 3A,B). In this case the nearly extended conformation did not prevent the binding of the same inhibitor to the other site, but probably in this second site the ligand, to avoid clashes with the ligand bound in site B of a symmetry-related molecule, must assume a bent conformation.

In all cases, the amino-ethyl-ureido chain of compounds 4-substitued did not form any direct H-bond or hydrophilic interaction with the enzyme, and eventually some were indirect mediated by water molecules.

## 3. Conclusions

Herein we reported a series of benzenesulfonamides incorporating ureido moieties. Compounds **16-35** were assayed in vitro as inhibitors of four hCA isoforms of pharmacological relevance, the cytosolic isoforms hCA I, II and the tumor associated transmembrane isoform hCA IX and XII. Interestingly the in vitro results showed a preferential inhibition of the cytosolic isoform hCA II and most potent inhibitors of this isoform were **18**, **19**, **24** and **34** with low nanomolar inhibition constants. We determined the binding modes of compounds **18, 19, 24, 26, 29** and **34** within adduct of hCA I isoform by means of X-ray crystallography techniques 

## 4. Experimental Protocols

### 4.1. Chemistry

All the reagents and solvents used in this study were purchased from Sigma-Aldrich. Moisture sensitive reactions were performed under nitrogen flow using anhydrous solvents and dried glassware. Nuclear magnetic resonance (^1^H-NMR, ^13^C-NMR and ^19^F-NMR) spectra were recorded on a Bruker Avance III 400 MHZ spectrometer using deuterated DMSO as a solvent and TMS (tetramethylsaline) as an internal standard. Chemical shifts (δ) are reported in parts per million (ppm) scale and coupling constants (*J* values) are given in Hertz (Hz). Splitting patterns are designated by: s (singlet); d (doublet); t (triplet); q (quartet); m (multiplet); brs (broad singlet) and dd (doublet of doublets). The exchangeable protons (NH and OH) were confirmed by D_2_O. Analytical thin-layer chromatography (TLC) was carried out on Merck Silica gel F-254 plates. Purification was carried out through crystallization and flash chromatography. Methanol/Dicholoromethane were used as a mobile phase (eluent) and Merck Silica gel 60 (230-400 mesh ASTM) as a stationary phase during flash chromatography. The high-resolution mass spectrometry (HRMS) analysis was performed with a Thermo Finnigan LTQ Orbitrap mass spectrometer coupled with an electrospray ionization source (ESI). The analysis was carried out in positive ion mode [M+H]^+^, and it was used a proper dwell time acquisition to achieve 60,000 units of resolution at full width at half maximum (fwhm). Melting points were determined in open capillary tubes with a Gallenkamp MPD350.BM3.5 apparatus and are uncorrected.

1. General Procedure for the Synthesis of Compound 5a-b

2. Synthesis of intermediates 4a–b. A Solution of **2a**–**b** (2.5 g, 1.0 eq) was treated with tert-butyl (2-aminoethyl) carbamate (1.0 eq) in acetonitrile (10 mL) and refluxed until the consumption of the starting materials (TLC monitoring). The reaction mixture was cooled to room temperature (r.t.) rt. Obtained precipitate was filtered-off, washed with Et_2_O (3 × 5 mL) and dried under vacuum to afford **4a**–**b** as a white solid.

3. tert-butyl (2-(3-(4-sulfamoylphenyl)ureido)ethyl)carbamate (4a): white solid; yield 80%; mp 173–174 °C; δ_H_ (400 MHz, DMSO-d_6_) 1.42 (9H, s), 3.05 (2H, q, J 5.8), 3.17 (2H, q, J 5.8), 6.33 (1H, t, J 5.8), 6.89 (2H, t, J 5.8), 7.17 (2H, s, exchange with D_2_O, SO_2_NH_2_), 7.56 (2H, d, J 8.8) 7.70 (2H, d, J 8.8), 8.97 (1H, s, exchange with D_2_O, NH); δ_C_ (100 MHz, DMSO-d_6_) 29.1, 40.0, 41.1, 78.5, 117.7, 127.6, 136.9, 144.5, 155.8, 156.6.

4. tert-butyl (2-(3-(3-sulfamoylphenyl)ureido)ethyl)carbamate (4b): white solid; yield 79%; mp 160–161 °C; TLC R_f_ 0.1 (EtOAc/n-hexane 70% *v*/*v*); δ_H_ (400 MHz, DMSO-d_6_) 1.42 (9H, s), 3.05 (2H, q, J 5.8), 3.17 (2H, q, J 5.8), 6.25 (1H, t, J 5.8), 6.89 (1H, t, J 5.8), 7.31 (2H, s, exchange with D_2_O, SO_2_NH_2_), 7.37 (1H, d, J 7.8), 7.43 (1H, t, 7.8), 7.55 (1H, d, J 7.8), 8.01 (1H, d, J 7.8), 8.90 (1H, s, exchange with D_2_O, NH); δ_C_ (100 MHz, DMSO-d_6_) 29.1, 41.2, 78.6, 115.4, 118.9, 121.3, 130.1, 141.9, 145.5, 155.9, 156.6.

5. Synthesis of intermediates 5**a**–**b**. A solution of **4a**–**b** (1 eq) in DCM was treated with fuming trifluoroacetic acid (6 eq), the reaction continued until the consumption of the starting material (TLC monitoring). Then the solvents were removed under reduced pressure to obtain a residue that was triturated from Et_2_O to obtain intermediates as **5a**–**b** as white solid.

6. 2-(3-(4-sulfamoylphenyl)ureido)ethan-1-aminium 2,2,2-trifluoroacetate (5a): white solid; yield 74%; mp 165–166 °C; δ_H_ (400 MHz, DMSO-d_6_) 2.95 (2H, m), 3.37 (2H, m), 6.94 (1H, t, J 5.8), 7.19 (2H, s, exchange with D_2_O, SO_2_NH_2_), 7.60 (2H, d, J 8.8) 7.71 (2H, d, J 8.8), 8.88 (3H, s, exchange with D_2_O, NH_3_), 9.45 (1H, s, exchange with D_2_O, NH); δ_C_ (100 MHz, DMSO-d_6_) 38.1, 40.2, 117.5, 127.6, 137.2, 144.5, 156.3; δ_F_ (376 MHz, DMSO-d_6_) -73.8.

7. 2-(3-(3-sulfamoylphenyl)ureido)ethan-1-aminium 2,2,2-trifluoroacetate (5b): white solid; yield 69%; mp 158–159 °C; δ_H_ (400 MHz, DMSO-d_6_) 2.96 (2H, m), 3.37 (2H, m), 6.87 (1H, t, J 6.8, exchange with D_2_O, NH), 7.33 (2H, s, exchange with D_2_O, SO_2_NH_2_), 7.39-7.47 (2H, m), 7.57 (1H, d, J 8), 7.88 (3H, s, exchange with D_2_O, NH_3_), 8.08 (1H, m), 9.36 (1H, s, exchange with D_2_O, NH); δ_C_ (100 MHz, DMSO-d_6_) 38.2, 40.2, 115.6, 119.2, 121.6, 130.2, 141.8, 145.5, 156.5; δ_F_ (376 MHz, DMSO-d_6_) -73.6. 

8. General Procedure for the Synthesis of compound 16-35. A solution of **5a-b** (0.2 g, 1.0 eq) in dry MeOH was treated with Et_3_N (1 eq) then the reaction mixture was cooled to 0 °C and benzaldehyde **6-15** (1 eq) was added. The reaction was continued for 2 h at 0 °C, followed by addition of sodium borohydride (4 eq) and reaction continued until the consumption of starting materials (TLC monitoring). The solvents were removed under reduced pressure and the title compounds were either obtained from filtration of the precipitates formed after addition of 5-10 mL of ice-cold water, followed by washing with Et_2_O (3 × 5 mL) (21, 26, 33) or extracted from ethyl acetate. In the latter the combined organic layers were washed with H_2_O (3 × 20 mL), dried over Na_2_SO_4_, filtered and concentrated in vacuum to give a residue that was crystallized from EtOH-Water (1:1) (**17**, **22**, **25**, **34**) or purified by silica gel column chromatography by eluting with 10%MeOH in DCM as the mobile phase (**16**, **18**–**20**, **23**–**24**, **27**–**30**, **35**).

9. 4-(3-(2-(benzylamino)ethyl)ureido) benzenesulfonamide (16): white solid; yield 41%; mp 176–177 °C; TLC R_f_ 0.12 (MeOH/DCM 10% *v*/*v*); δ_H_ (400 MHz, DMSO-d_6_) 2.22 (1H, t, J 5.8, exchange with D_2_O, NH), 2.63 (2H, q, J 5.8), 3.22 (2H, q, J 5.8), 3.74 (2H, d, J 5.8), 6.49 (1H, brt, J 5.8, exchange with D_2_O, NH), 7.15 (2H, s, exchange with D_2_O, SO_2_NH_2_), 7.25 (1H, t, J 7.2), 7.32-7.39 (4H, m), 7.56 (2H, d, J 8.8), 7.69 (2H, d, J 8.8), 9.15 (1H, s, exchange with D_2_O, NH); δ_C_ (100 MHz, DMSO-d_6_) 40.1, 49.3, 53.6, 117.6, 127.4, 127.6, 128.9, 129.0, 137.0, 141.8, 144.7, 155.9; *m*/*z* (ESI positive) 349.1 [M+H]^+^.

10. 4-(3-(2-((4-chlorobenzyl)amino)ethyl)ureido) benzenesulfonamide (17): white solid; yield 44%; mp 20–-205 °C; TLC R_f_ 0.10 (MeOH/DCM 10% *v*/*v*); δ_H_ (400 MHz, DMSO-d_6_) 2.60 (2H, t, J 6), 3.21 (2H, q, J 6), 3.73 (2H, brs), 6.42 (1H, t, J 6, exchange with D_2_O, NH), 7.19 (2H, s, exchange with D_2_O, SO_2_NH_2_), 7.40 (4H, m), 7.56 (2H, d, J 8.8), 7.69 (2H, d, J 8.8), 9.09 (1H, s, exchange with, D_2_O, NH); δ_C_ (100 MHz, DMSO-d_6_) 39.8, 49.2, 52.8, 117.6, 127.7, 128.9, 130.7, 131.9,136.8, 140.9, 144.6, 155.8; *m*/*z* (ESI positive) 383.0 [M+H]^+^.

11. 4-(3-(2-((4-fluorobenzyl)amino)ethyl)ureido) benzenesulfonamide (18): Pale yellow solid; yield 30%; mp 191–192 °C; TLC R_f_ 0.10 (MeOH/DCM 10% *v*/*v*); δ_H_ (400 MHz, DMSO-d_6_) 2.75 (2H, t, J 5.8), 3.26 (2H, q, J 5.8), 3.89 (2H, brs), 6.41 (1H, t, J 5.8, exchange with D_2_O, NH), 7.19-7.24 (4H, m, 2H exchange with D_2_O, SO_2_NH_2_), 7.48 (2H, m), 7.55 (2H, d, J 8.8), 7.70 (2H, d, J 8.8), 9.16 (1H, s, exchange with D_2_O, NH); δ_C_ (100 MHz, DMSO-d_6_) 38.8, 48.6, 51.8, 115.9 (d, ^2^J_C-F_ 21), 117.7, 127.7, 131.7 (d, ^3^J_C-F_ 8), 134.6 (d, ^4^J_C-F_ 3), 136.9, 144.6, 156.1, 162.5 (d, ^1^J_C-F_ 242), δ_F_ (376 MHz, DMSO-d_6_) -115.1 (1F, s); *m*/*z* (ESI positive) 367.1 [M+H]^+^.

12. 4-(3-(2-((2-fluorobenzyl)amino)ethyl)ureido) benzenesulfonamide (19): White solid; yield 41%; mp 156–157 °C; TLC R_f_ 0.10 (MeOH/DCM 10% *v*/*v*); δ_H_ (400 MHz, DMSO-d_6_) 2.71 (2H, t, J 5.8), 3.26 (2H, q, J 5.8), 3.86 (2H, brs), 6.42 (1H, t, J 5.8, exchange with D_2_O, NH), 7.19 (2H, s, exchange with D_2_O, SO_2_NH_2_), 7.19-7.24 (2H, m), 7.35 (1H, m), 7.52-7.58 (3H, m), 7.70 (2H, d, J 8.8), 9.09 (1H, s, exchange with D_2_O, NH); δ_C_ (100 MHz, DMSO-d_6_) 39.4, 46.1 (d, J 3.3), 49.1, 116.0 (d, J_C-F_ 22), 116.1, 117.6, 125.2 (d, J_C-F_ 3.5), 127.6, 130.0 (d, J_C-F_ 8.7), 131.6 (d, J_C-F_ 4.4), 136.9, 144.7, 155.9, 161.4 (d, J_C-F_ 243); δ_F_ (376 MHz, DMSO-d_6_) -118.9 (1F, s); *m*/*z* (ESI positive) 367.1 [M+H]^+^.

13. 4-(3-(2-((4-hydroxybenzyl)amino)ethyl)ureido) benzenesulfonamide (20): Pale yellow solid; yield 28%; mp 134–135 °C; TLC R_f_ 0.10 (MeOH/DCM 10% *v*/*v*); δ_H_ (400 MHz, DMSO-d_6_) 2.60 (2H, t, J 6), 3.20 (2H, q, J 6), 3.62 (2H, brs), 6.39 (1H, t, J 6, exchange with D_2_O, NH), 6.72 (2H, d, J 8.5), 7.15 (4H, m, 2H exchange with D_2_O, SO_2_NH_2_), 7.55 (2H, d, J 8.8), 7.69 (2H, d, J 8.8), 9.08 (1H, s, exchange with D_2_O, NH), 9.23 (1H, s, exchange with D_2_O, OH); δ_C_ (100 MHz, DMSO-d_6_) 40.1, 49.2, 53.3, 115.9, 117.7, 127.8, 130.2, 131.8, 136.9, 144.8, 155.9, 157.0; *m*/*z* (ESI positive) 365.1 [M+H]^+^.

14. 4-(3-(2-((4-nitrobenzyl)amino)ethyl)ureido) benzenesulfonamide (21): White solid, yield 38%; mp 191–192 °C; TLC R_f_ 0.11 (MeOH/DCM 10% *v*/*v*); δ_H_ (400 MHz, DMSO-d_6_) 2.63 (2H, t, J 6.2), 3.23 (2H, q, J 6.2), 3.88 (2H, brs), 6.41 (1H, t, J 6.2, exchange with D_2_O, NH), 7.19 (2H, s, exchange with D_2_O, SO_2_NH_2_), 7.55 (2H, d, J 8.5), 7.68 (4H, m), 8.22 (2H, d, J 7.9), 9.06 (1H, s, exchange with D_2_O, NH); δ_C_ (100 MHz, DMSO-d_6_) 40.1, 49.4, 52.9, 117.7, 124.2, 127.7, 129.8, 136.9, 144.7, 147.2, 150.4, 155.8; *m*/*z* (ESI positive) 394.1 [M+H]^+^.

15. 4-(3-(2-((4-(dimethylamino)benzyl)amino)ethyl)ureido) benzenesulfonamide (22): White solid, yield 24%; mp 162–163 °C; TLC R_f_ 0.13 (MeOH/DCM 10% *v*/*v*); δ_H_ (400 MHz, DMSO-d_6_) 2.60 (2H, t, J 6), 2.89 (6H, s), 3.21 (2H, q, J 6), 3.62 (2H, brs), 6.41 (1H, t, J 6, exchange with D_2_O, NH), 6.70 (2H, d, J 8.5), 7.18 (4H, m, 2H exchange with D_2_O, SO_2_NH_2_), 7.56 (2H, d, J 8.8), 7.70 (2H, d, J 8.8), 9.09 (1H, s, exchange with D_2_O, NH); δ_C_ (100 MHz, DMSO-d_6_) 40.0, 41.3, 49.1, 53.2, 113.2, 117.6, 127.6, 129.2, 129.7, 136.8, 144.7, 150.4, 155.9; *m*/*z* (ESI negative) 436.0 [M+HCOO]^−^

16. 4-(3-(2-((2-methoxy-4-nitrobenzyl)amino)ethyl)ureido)benzenesulfonamide (23): Pale yellow solid, yield 31%; mp 199–200 °C; TLC R_f_ 0.12 (MeOH/DCM 10% *v*/*v*); δ_H_ (400 MHz, DMSO-d_6_) 2.66 (2H, t, J 6), 3.24 (2H, q, J 6), 3.81 (2H, brs), 3.96 (3H, s), 6.38 (1H, t, J 6, exchange with D_2_O, NH), 7.18 (2H, s, exchange with D_2_O, SO_2_NH_2_), 7.56 (2H, d, J 8.9), 7.70 (3H, m), 7.77 (1H, d, J 2.1), 7.88 (1H, dd, J 2.1, 8.3), 9.03 (1H, s, exchange with D_2_O, NH); δ_C_ (100 MHz, DMSO-d_6_) 39.7, 47.5, 49.4, 57.0, 106.1, 116.3, 117.6, 127.6, 130.3, 137.0, 144.7, 148.5, 156.0, 158.2; *m*/*z* (ESI positive) 424.1 [M+H]^+^.

17. 4-(3-(2-((4-bromo-2-hydroxybenzyl)amino)ethyl)ureido) benzenesulfonamide (24): White solid, yield 23%; mp 164–165 °C; TLC R_f_ 0.11 (MeOH/DCM 10% *v*/*v*); δ_H_ (400 MHz, DMSO-d_6_) 2.71 (2H, t, J 5.8), 3.27 (2H, q, J 5.8), 3.85 (2H, brs), 6.44 (1H, t, J 5.8, exchange with D_2_O, NH), 6.97 (2H, m), 7.17 (3H, m, 2H exchange with D_2_O, SO_2_NH_2_), 7.56 (2H, d, J 8.8), 7.69 (2H, d, J 8.8), 9.07 (1H, s, exchange with D_2_O, NH); δ_C_ (100 MHz, DMSO-d_6_) 39.4, 48.9, 49.4, 117.7, 118.8, 121.2, 122.2, 124.5, 127.7, 131.7, 137.0, 144.7, 156.0, 159.0; *m*/*z* (ESI positive) 442.9 [M+H]^+^.

18. 4-(3-(2-(([1,1′-biphenyl]-4-ylmethyl)amino)ethyl)ureido) benzenesulfonamide (25): White solid, yield 37%; mp 197–198 °C; TLC R_f_ 0.13 (MeOH/DCM 10% *v*/*v*); δ_H_ (400 MHz, DMSO-d_6_) 2.66 (2H, t, J 6.1), 3.24 (2H, q, J 6.1), 3.79 (2H, brs), 6.41 (1H, brt, J 6.1, exchange with D_2_O, NH), 7.18 (2H, s, exchange with D_2_O, SO_2_NH_2_), 7.38 (1H, t, J 7.4), 7.49 (4H, m), 7.56 (2H, d, J 8.8), 7.63-7.70 (6H, m), 9.07 (1H, s, exchange with D_2_O, NH); δ_C_ (100 MHz, DMSO-d_6_) 39.6, 49.3, 53.3, 117.7, 127.4, 127.5, 127.7, 128.2, 129.5, 129.8, 136.9, 139.4, 141.0, 141.1, 144.7, 155.9; *m*/*z* (ESI positive) 425.1 [M+H]^+^.

19. 3-(3-(2-(benzylamino)ethyl)ureido)benzenesulfonamide (26): Pale yellow solid, yield 16%; mp 109–110 °C; TLC R_f_ 0.12 (MeOH/DCM 10% *v*/*v*); δ_H_ (400 MHz, DMSO-d_6_) 2.63 (2H, t, J 6), 3.22 (2H, q, J 6), 3.75 (2H, brs), 6.29 (1H, t, J 6, exchange with D_2_O, NH), 7.25 (1H, t, J 7), 7.32-7.44 (8H, m, 2H exchange with D_2_O, SO_2_NH_2_), 7.54 (1H, d, J 7.9), 8.00 (1H, brt, J 1.6), 8.97 (1H, s, exchange with D_2_O, NH); δ_C_ (100 MHz, DMSO-d_6_) 39.8, 49.3, 53.6, 115.3, 118.8, 121.2, 127.5, 128.9, 129.0, 130.1, 141.7, 142.0, 145.5, 156.0; *m*/*z* (ESI positive) 349.1 [M+H]^+^.

20. 3-(3-(2-((4-chlorobenzyl)amino)ethyl)ureido) benzenesulfonamide (27): Pale yellow solid, yield 10%; mp 102–103 °C; TLC R_f_ 0.11 (MeOH/DCM 10% *v*/*v*); δ_H_ (400 MHz, DMSO-d_6_) 2.67 (2H, t, J 6), 3.24 (2H, q, J 6), 3.79 (2H, brs), 6.32 (1H, t, J 6, exchange with D_2_O, NH), 7.32 (2H, s, exchange with D_2_O, SO_2_NH_2_), 7.36-7.44 (6H, m), 7.54 (1H, d, J 7.8), 8.00 (1H, s), 8.98 (1H, s, exchange with D_2_O, NH); δ_C_ (100 MHz, DMSO-d_6_) 39.7, 49.2, 52.5, 115.5, 119.0, 121.4, 129.1, 130.2, 131.0, 132.4, 139.8, 142.0, 145.6, 156.2; *m*/*z* (ESI positive) 383.1 [M+H]^+^.

21. 3-(3-(2-((4-fluorobenzyl)amino)ethyl)ureido) benzenesulfonamide (28): Pale yellow solid, yield 35%; mp 104–105 °C; TLC R_f_ 0.11 (MeOH/DCM 10% *v*/*v*); δ_H_ (400 MHz, DMSO-d_6_) 2.68 (2H, t, J 6), 3.25 (2H, q, J 6), 3.80 (2H, brs), 6.38 (1H, t, J 6, exchange with D_2_O, NH), 7.18 (2H, m), 7.33 (2H, s, exchange with D_2_O, SO_2_NH_2_), 7.37-7.47 (4H, m), 7.55 (1H, d, J 8.0), 8.02 (1H, d, J 1.8), 9.05 (1H, s, exchange with D_2_O, NH); δ_C_ (100 MHz, DMSO-d_6_) 39.6, 49.1, 52.4, 115.4, 115.8 (d, ^2^J_C-F_ 23), 118.9, 121.3, 130.2, 131.1 (d, ^3^J_C-F_ 8), 136.8, 142.0, 145.5, 156.1, 162.2 (d, ^1^J_C-F_ 240); δ_F_ (376 MHz, DMSO-d_6_) -116.1 (1F, s); *m*/*z* (ESI positive) 367.1 [M+H]^+^.

22. 3-(3-(2-((2-fluorobenzyl)amino)ethyl)ureido) benzenesulfonamide (29): Pale yellow solid, yield 15%; mp 155–156 °C; TLC R_f_ 0.12 (MeOH/DCM 10% *v*/*v*); δ_H_ (400 MHz, DMSO-d_6_) 2.72 (2H, t, J 6), 3.27 (2H, q, J 6), 3.87 (2H, brs), 6.34 (1H, t, J 6, exchange with D_2_O, NH), 7.22 (2H, m), 7.32 (2H, s, exchange with D_2_O, SO_2_NH_2_), 7.34-7.45 (3H, m), 7.55 (2H, m), 8.01 (1H, brt, J 1.8), 9.00 (1H, s, exchange with D_2_O, NH); δ_C_ (100 MHz, DMSO-d_6_) 39.5, 46.2 (d, J 3.4), 49.2, 115.5, 116.0 (d, J 22), 119.0, 121.4, 125.3 (d, J_C-F_ 3.6), 126.9 (d, J 14.7), 130.1 (d, J 8), 130.2, 131.7 (d, J 5), 142.0, 145.5, 156.2, 161.3 (d, J 243); δ_F_ (395 MHz, DMSO-d_6_) -118.7 (1F, s); *m*/*z* (ESI positive) 367.1 [M+H]^+^.

23. 3-(3-(2-((4-hydroxybenzyl)amino)ethyl)ureido) benzenesulfonamide (30): Orange solid, yield 15%; mp 133–134 °C; TLC R_f_ 0.10 (MeOH/DCM 10% *v*/*v*); δ_H_ (400 MHz, DMSO-d_6_) 2.59 (2H, t, J 6), 3.20 (2H, q, J 6), 3.61 (2H, brs), 6.36 (1H, brt, J 6, exchange with D_2_O, NH), 6.72 (2H, d, J 8.4), 7.15 (2H, d, J 8.4), 7.34-7.44 (3H, m, 2H exchange with D_2_O, SO_2_NH_2_), 7.42 (1H, t, J 7.9), 7.53 (1H, d, J 7.9), 8.01 (1H, brt, J 1.8), 9.06 (1H, s, exchange with D_2_O, NH), 9.27 (1H, s, exchange with D_2_O, OH); δ_C_ (100 MHz, DMSO-d_6_) 39.2, 49.5, 53.5, 116.5, 116.7, 120.3, 123.0, 131.3, 132.0, 142.2, 145.6, 157.3, 157.4; *m*/*z* (ESI positive) 365.1 [M+H]^+^.

24. 3-(3-(2-((4-nitrobenzyl)amino)ethyl)ureido) benzenesulfonamide (31): Pale yellow solid, yield 10%; mp 109–110 °C; TLC R_f_ 0.11 (MeOH/DCM 10% *v*/*v*); δ_H_ (400 MHz, DMSO-d_6_) 2.62 (2H, t, J 6), 3.24 (2H, q, J 6), 3.91 (2H, brs), 6.33 (1H, t, J 6, exchange with D_2_O, NH), 7.33-7.45 (4H, m, 2H, s, exchange with D_2_O, SO_2_NH_2_), 7.54 (1H, d, J 8.0), 7.68 (2H, d, J 8.6), 8.01 (1H, t, J 2), 8.23 (2H, d, J 8.6), 8.99 (1H, s, exchange with D_2_O, NH); δ_C_ (100 MHz, DMSO-d_6_) 39.8, 49.4, 52.8, 115.6, 119.3, 121.7, 124.5, 130.3, 130.5, 142.0, 145.5. 147.6, 149.9, 156.3; *m*/*z* (ESI positive) 394.0 [M+H]^+^.

25. 3-(3-(2-((4-(dimethylamino)benzyl)amino)ethyl)ureido) benzenesulfonamide (32): Pale brown solid, yield 10%; mp 101–103 °C; TLC R_f_ 0.13 (MeOH/DCM 10% *v*/*v*); δ_H_ (400 MHz, DMSO-d_6_) 2.64 (2H, t, J 5.8), 2.89 (6H, s), 3.23 (2H, q, J 5.8), 3.66 (2H, brs), 6.31 (1H, t, J 5.8, exchange with D_2_O, NH), 6.71 (2H, d, J 8.6), 7.19 (2H, d, J 8.6), 7.32-7.38 (3H, m, 2H exchange with D_2_O, SO_2_NH_2_), 7.43 (1H, t, J 7.8), 7.54 (1H, d, J 7.8), 8.00 (1H, t, J 1.8), 9.00 (1H, s, exchange with D_2_O, NH); δ_C_ (100 MHz, DMSO-d_6_) 39.8, 41.2, 48.9, 52.9, 113.2, 115.4, 118.9, 121.3, 129.9, 130.2, 142.0, 145.5, 150.5, 156.1; *m*/*z* (ESI negative) 436.0 [M+HCOO]^−^.

26. 3-(3-(2-((2-methoxy-4-nitrobenzyl)amino)ethyl)ureido)benzenesulfonamide (33): Yellow solid, yield 42%; mp 103–105 °C; TLC R_f_ 0.12 (MeOH/DCM 10% *v*/*v*); δ_H_ (400 MHz, DMSO-d_6_) 2.66 (2H, t, J 5.8), 3.25 (2H, q, J 5.8), 3.81 (2H, brs), 3.96 (3H, s), 6.33 (1H, t, J 6.8, exchange with D_2_O, NH), 7.33 (2H, s, exchange with D_2_O, SO_2_NH_2_), 7.40-7.48 (2H, m), 7.58 (1H, m), 7.76 (1H, d, J 8.4), 7.89 (1H, d, J 2.2), 7.97 (1H, dd, J 8.4, 2.2), 8.01 (1H, t, J 2), 8.99 (1H, s, exchange with D_2_O, NH); δ_C_ (100 MHz, DMSO-d_6_) 40.1, 47.7, 49.6, 57.0, 105.9, 115.4, 116.3, 118.9, 121.3, 129.8, 130.2, 138.1, 142.0, 145.5, 148.1, 156.0, 158.1; *m*/*z* (ESI positive) 424.1 [M+H]^+^.

27. 3-(3-(2-((4-bromo-2-hydroxybenzyl)amino)ethyl)ureido) benzenesulfonamide (34): Pale yellow solid, yield 13%, mp 113–114 °C; TLC R_f_ 0.11 (MeOH/DCM 10% *v*/*v*); δ_H_ (400 MHZ, DMSO-d_6_) 2.65 (2H, t, J 6), 3.22 (2H, q, J 6), 3.78 (2H, brs), 6.30 (1H, t, J 6, exchange with D_2_O, NH), 6.95 (2H, m), 7.12 (1H, d, J 7.8), 7.32 (2H, s, exchange with D_2_O, SO_2_NH_2_), 7.35-7.38 (1H, m), 7.43 (1H, t, J 7.8), 7.55 (1H, m), 8.01 (1H, t, J 2), 8.94 (1H, s, exchange with D_2_O, NH): δ_C_ (100 MHz, DMSO-d_6_) 39.7, 49.1, 49.9, 115.4, 118.8, 118.9, 120.8, 121.3, 122.1, 125.2, 130.1, 131.3, 141.9, 145.5. 155.9, 159.1; *m*/*z* (ESI negative) 440.9 [M-H]^−^.

28. 3-(3-(2-(([1,1’-biphenyl]-4-ylmethyl)amino)ethyl)ureido) benzenesulfonamide (35): White solid, yield 17%; mp 116–117 °C; TLC R_f_ 0.13 (MeOH/DCM 10% *v*/*v*); δ_H_ (400 MHz, DMSO-d_6_) 2.76 (2H, t, J 6), 3.30 (2H, q, J 6), 3.90 (2H, brs), 6.39 (1H, t, J 6, exchange with D_2_O, NH), 7.33 (2H, s, exchange with D_2_O, SO_2_NH_2_), 7.36-7.45 (3H, m), 7.48-7.57 (5H, m), 7.66-7.70 (4H, m), 8.02 (1H, t, J 2), 9.06 (1H, s, exchange with D_2_O, NH); δ_C_ (100 MHz, DMSO-d_6_) 39.4, 49.1, 52.7, 115.7, 119.4, 121.8, 127.7, 128.6, 130.2, 130.3, 130.5, 132.7, 138.8, 140.3, 141.1, 141.9, 145.5, 156.4; *m*/*z* (ESI positive) 425.1 [M+H]^+^.

### 4.2. CA Inhibition

An applied photophysics stopped-flow instrument measuring CA catalyzed CO_2_ hydration by a spectrophotometric method [38] was employed for obtaining the inhibition constants of the new compounds reported here. The used indicator was phenol red (0.2 mM), the buffer was HEPES (20 mM, pH 7.5) and ionic strength was maintained constant by the use of 20 mM Na_2_SO_4_. CO_2_ hydration rates were followed for 10–100 s, using CO_2_ concentrations in the range of 1.7–17 mM. Each inhibitor was tested in triplicate, in serial dilutions starting from 0.01 to 0.1 mM. An incubation time of 15 min between inhibitor and enzyme has been used, as reported earlier, in order to be sure that the enzyme–inhibitor complex has been formed [2,25,27,39,40,41,42,43,44,45,46,47,48,49,50]. The inhibition constants were obtained by using the Cheng–Prusoff equation [39,51,52,53] and are the mean from three determinations. The employed CA isoforms were recombinant proteins obtained by us as reported in earlier publications [39,51,52].

### 4.3. Crystallization, Data Collection and Structure Determination

Crystals of hCAI were obtained using the sitting drop vapor diffusion method in 96 well SWISSCI MRC 2 plate. A solution of 0.5 µL of 10 mg ml^−1^ of hCAI (purchased from Sigma-Aldrich) in 50 mM Tris-HCl pH 8.7 was mixed with 0.5 µL of a solution of 200 mM Na-acetate, 30% PEG 4000, 100 mM Tris-HCl pH 9. Single crystals were obtained in about one week of incubation at 20 °C. Crystals of hCAI in complex with inhibitors (compounds **18**, **19**, **24**, **26**, **29** and **34**) were prepared by soaking 0.2 µL of 10 mM inhibitor solution (150 mM NaCl, 10% DMSO, 50 mM Tris pH 7) in a drop containing crystals of the hCAI apoenzyme. Frames with an oscillation of 0.1° each were collected at the I03 MX beamline of Diamond Light Source (Didcot, UK). All the crystals belong to the space group P2_1_2_1_2_1_, with two monomers in the asymmetric unit. Datasets were processed automatically with the xia2 expert system (https://xia2.github.io/index.html) and scaled with Aimless [54]. The space group resulted in being isomorphous with the native apo hCA I and the structure of hCA I in a complex with the inhibitor famotidine (PDB ID 6g3v) was used as a starting points for the refinement [55]. The structure was refined using the software package Phenix [56]. After a few cycles, a clear density in the active site was clearly visible and a model of the appropriate inhibitor was manually fitted and refined. Map visualization and manual adjustment of the models were performed using the Coot graphic interface [57]. Statistics on data collection and refinement are reported in Supporting Information Table S1.

## Figures and Tables

**Table 1:** Inhibition data of human CA isoforms hCA I, II, IX and IXI with 16-35 reported here and the standard sulfonamide inhibitor acetazolamide (AAZ) by a stopped flow CO_2_ hydrase assay [38].

K_I_ (nM) *					
Compound	R	hCA I	hCA II	hCA IX	hCA XII
**16**		552.4	350.1	1350	54.5
**17**	4-Cl	582.1	126.9	458.3	32
**18**	4-F	724.7	9.2	30.1	53.2
**19**	2-F	24.6	3.9	20.3	58.3
**20**	4-OH	781	267	974.2	75.8
**21**	4-NO_2_	526.9	96.1	317.1	77.5
**22**	4-Me_2_N	1506	73.2	342.8	264.1
**23**	4-NO_2_, 2-MeO	788.4	82.4	290.6	53.6
**24**	4-Br, 2-OH	138.4	5.3	115.6	97.6
**25**	4-C_6_H_5_	781.7	63.2	1150	63.7
**26**	-	928.3	76.3	>10,000	772.1
**27**	4-Cl	702.4	36.9	3378	228.1
**28**	4-F	867.5	53.8	4212	71.3
**29**	2-F	728	33.4	>10,000	478.8
**30**	4-OH	6250	166.4	3511	317.1
**31**	4-NO_2_	5851	168.4	2325	60.7
**32**	4-Me_2_N	8906	564.9	1199	78
**33**	4-NO_2,_ 2-MeO	>10,000	90.2	2606	53.1
**34**	4-Br, 2-OH	653.8	2.8	1915	7.2
**35**	4-C_6_H_5_	>10,000	456.3	>10,000	688
**AAZ**		250	12.1	25.8	5.7

* Mean from 3 different assays, by a stopped flow technique (errors were in the range of ± 5%–10% of the reported values).

## Supplementary Materials

The following are available online at https://www.mdpi.com/1422-0067/21/7/2560/s1, Figure S1: title, Table S1: title, Video S1: title.
