# Supplementary material for: Benzylaminoethyureido-Tailed Benzenesulfonamides: Design, Synthesis, Kinetic and X-ray Investigations on Human Carbonic Anhydrases
Source: Int J Mol Sci. 2020 Apr 7;21(7):2560. doi: 10.3390/ijms21072560 (PMC7177897; doi:10.3390/ijms21072560)
Supplement: Supplementary file 1 [file ijms-21-02560-s001.pdf]

Supporting Information for

Benzylaminoethyureido-tailed benzenesulfonamides: Design, synthesis, kinetic and X-ray investigations on human Carbonic anhydrases

Majid Ali,[1,2,3] Murat Bozdag,[1,*] Umar Farooq,[3] Andrea Angeli,[1] and Fabrizio Carta[1], Paola Berto [2], Giuseppe Zanotti [2,*], Claudiu T. Supuran[1]

[1] Università degli Studi di Firenze, Dipartimento Neurofarba, Sezione di Scienze Farmaceutiche, Polo Scientifico, Via Ugo Schiff 6, 50019 Sesto Fiorentino, Florence, Italy

[2] Università di Padova, Department of Biomedical Sciences, Via Ugo Bassi 58/B, Padua, 35131, Italy.

[3] Department of Chemistry, COMSATS University Islamabad, Abbottabad Campus, 22060, KPK, Pakistan

Index

Statistics on data collection, processing and structure determination S2-S3

	18	19	24	26	29	34
**Data collection**						
Cell parameters (a, b, c, Å)	62.56, 71.66, 121.83	62.60, 71.65, 121.69	62.07, 70.89, 121.51	62.84, 70.98, 120.80	62.51, 70.85, 121.56	62.57, 71.38, 121.40
Resolution (Å)	62.56 – 1.53<br>(1.57 – 1.53)	62.60 – 1.54<br>(1.58 – 1.54)	61.22 – 1.44<br>(1.48 – 1.44)	62.85 – 1.55<br>(1.59 – 1.55)	62.51 – 1.66<br>(1.70 – 1.66)	61.5 – 1.37<br>(1.41-1.37)
Completeness (%)	100.0 (100.0)	100.0 (100.0)	100.0 (100.0)	100.0 (100.0)	100.0 (100.0)	99.9 (98.3)
Unique reflections	83442 (6072)	81860 (5955)	97700 (7186)	79244 (5757)	64678 (4718)	114640 (8252)
Multiplicity	13.3 (13.6)	13.2 (13.4)	13.2 (12.4)	13.1 (13.4)	13.2 (13.4)	12.5 (7.4)
Rmerge	0.091 (1.899)	0.103 (1.981)	0.148 (2.455)	0.083 (1.793)	0.144 (1.945)	0.063 (1.25)
Rpim	0.027 (0.556)	0.029 (0.557)	0.043 (0.735)	0.025(0.526)	0.042 (0.57)	0.019 (0.522)
<I/sigma(I)>	12.5 (1.2)	11.4 (1.2)	9.7 (1.1)	12.2 (1.1)	8.6 (1.2)	14.6 (1.0)
**Refinement**						
Rcryst/Rfree	0.1962/0.2184	0.1901/0.2141	0.1862/0.2213	0.2002 / 0.2241	0.1855 / 0.2209	0.1978/0.2184
Protein atoms/ligand / solvent atoms	4031/52/680	4031/52/718	4031/28/849	4031/50/610	4031/52/700	4031/54/739
**Geometry**						
Ramachandran favored/outliers (%)	96.86/0.0	96.47/0.0	97.06/0.0	96.86 /0.0	96.67/0.0	96.67/0.0
Rotamer outliers (%) /Overall score	0.0 / 1.24	0.00/1.24	0.22/1.20	0.22/1.24	0.22/1.23	0.22/1.32

r.m.s.d. on bond length (Å), angles (°)	0.008 / 0.99	0.009, 0.84	0.005, 0.96	0.007/0.85	0.006, 0.89	0.005, 0.84
**Ligand validation**						
Isotropic B value	41.1 (A)<br>38.8 (B)	41.0 (A)<br>36.5 (B)	31.6	37.8 (A)<br>31.5 (B)	39.0 (A)<br>34.8 (B)	31.7 (A)<br>47.0 (B)
Real-space Correlation Coefficient (RSCC)	0.92 (A)<br>0.96 (B)	0.95 (A)<br>0.96 (B)	0.913	0.95 (A)<br>0.97 (B)	0.93 (A)<br>0.97 (B)	0.94 (A)<br>0.83 (B)

Table S1. Statistics on data collection, processing and structure determination. In all cases, a rotation of 360° was applied, with a rotation/frame of 0.1°. The wavelength used was 0.97624 Å. Number in parentheses refer to the high-resolution shell. Data were kept at the maximum resolution compatible with a <I/sigma(I)> larger or equal to 1.
